# A novel role for SALL4 during scar-free wound healing in axolotl

**DOI:** 10.1038/npjregenmed.2016.16

**Published:** 2016-12-08

**Authors:** Jami R Erickson, Micah D Gearhart, Drew D Honson, Taylor A Reid, Melissa K Gardner, Branden S Moriarity, Karen Echeverri

**Affiliations:** 1 Department of Genetics, Cell Biology and Development, University of Minnesota, Minnesota, MN, USA; 2 Department of Pediatrics, University of Minnesota, Center for Genome Engineering, University of Minnesota, Masonic Cancer Center, University of Minnesota, Minnesota, MN USA

## Abstract

The human response to serious cutaneous damage is limited to relatively primitive wound healing, whereby collagenous scar tissue fills the wound bed. Scars assure structural integrity at the expense of functional regeneration. In contrast, axolotls have the remarkable capacity to functionally regenerate full thickness wounds. Here, we identified a novel role for SALL4 in regulating collagen transcription after injury that is essential for perfect skin regeneration in axolotl. Furthermore, we identify miR-219 as a molecular regulator of *Sall4* during wound healing. Taken together, our work highlights one molecular mechanism that allows for efficient cutaneous wound healing in the axolotl.

## Introduction

The skin is the largest organ of the human body and has several crucial functions to keep the body operational and healthy. It is a barrier to the outside world; it regulates metabolic functions and acts as a structural framework for the body. The skin is an organ that is in a perpetual state of change, constantly replacing the outermost layer of cells via division and differentiation of basal keratinocytes. Humans can easily repair minor tears to the skin but major skin injuries result in incompletely remodelled collagen in the wound bed, which manifests as part of a fibrotic scar.^1–6^

In contrast, the Mexican ‘Axolotl’ salamander is able to fully regenerate the skin after major wounding.^7–10^ Previous work shows that at the end of the wound healing process in axolotls collagen remodelling and wound bed closure is complete, and the skin is restored to normal functionality. Thus, a common hypothesis is that axolotl and human have differing molecular mechanisms of cutaneous wound healing that directs towards scar-free regeneration in axolotls versus reparative scar prone healing in humans.

Until now, some interesting differences in the wound healing processes between these two species have been established. For example, a major difference between axolotls and humans is the speed at which re-epithelialization of the wound occurs.^7,9–11^ In axolotls this process occurs directly after injury; whereby keratinocytes migrate over the fibrin clot to close the wound within 24 h. Once the wound is covered, the keratinocytes will start to proliferate to thicken the epidermis. In contrast, human keratinocytes at the leading edge of the wound will hyperproliferate and then migrate under the fibrin clot to close the wound ~ 1 week after wound formation. A second major difference between axolotls and humans is the timing at which collagen is deposited in the wound area. In humans, extracellular matrix (ECM) deposition by dermal fibroblasts begins between 2 to 5 days after injury, leading to scar formation by ~15 days after wounding. Comparatively, in axolotls, dermal fibroblasts are recruited to the injury site within 5 days after injury, but ECM deposition does not begin until 10–15 days after wounding. A third major difference between human and axolotl wound healing is the degree to which the ECM that has been remodelled at the end of the entire wound healing process. The major component of the ECM in both human and axolotl skin is collagen. A major driver of scar formation is the lack of remodelling of collagen to a basket-weave formation that is present in normal skin. In humans, at the end of the wound healing process collagen remains in thick aligned bundles, meanwhile in axolotl’s collagen is remodelled during the regeneration process and the skin returns to its normal functionality.^9,10^

To identify key molecular pathways that are necessary to drive scar-free wound healing in axolotl, we carried out transcriptional profiling at different time points during regeneration, and compared this to publically available human skin wound healing arrays. This approach allowed us to identify Sal-like 4 (Sall4) as a gene expressed early after wounding in axolotl. SALL4 is a transcription factor that is known for its role in maintaining ‘stemness’ of both induced pluripotent stem cells and embryonic stem cells.^12–17^ Mechanistically, SALL4 has been shown to interact with the transcription factors OCT4, NANOG and SOX2.^13,14,18–20^ In addition, SALL4 has been shown to be required for embryonic survival and development of multiple tissue lineages, and is differentially regulated during *Xenopus* and axolotl limb regeneration.^12,13,18,19,21–25^ Taken together, these studies suggest that SALL4 is part of the molecular mechanism responsible for maintaining cells in a less differentiated state.

In this study, we examine the role of SALL4 during the cutaneous wound healing process in axolotl. Thus, we identify a novel molecular mechanism whereby SALL4 in the axolotl regulates collagen production during dermal regeneration. That is an essential component of the molecular circuitry necessary for the ability of axolotls to heal cutaneous wounds scar-free.

## Results

### Identification of axolotl Sall4 gene

To identify genes that are differentially regulated during full thickness wound healing in axolotl, 2 mm punch biopsies were taken from uninjured animals and from animals at 2, 14 and 21 days post injury. RNA was extracted and used to perform transcriptional profiling with axolotl-specific microarrays. Expression values were calculated using a robust multi-array average for background correction and normalisation. Log2-fold changes were calculated for each injured sample relative to the uninjured control using a linear regression model. Of the 4,302 differentially expressed probes (adjusted *P* value <0.05), 808 probes were differentially expressed in the axolotl by twofold or more at 2 days post injury.

**Figure 1:**
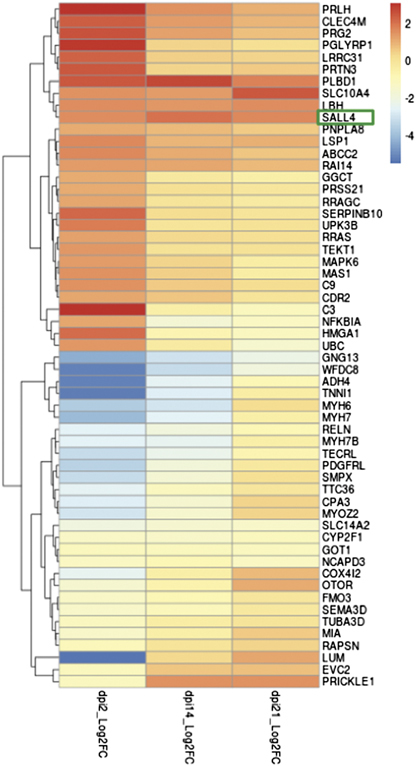
Transcriptional analysis of axolotl versus human mRNA profiles during wound healing. The heat map was generated based on significant differences observed in axolotl versus human skin after injury, this approach identified 57 genes that significantly differentially regulated in axolotl skin 2 days post injury but do not change in humans at this time point.

**Figure 2:**
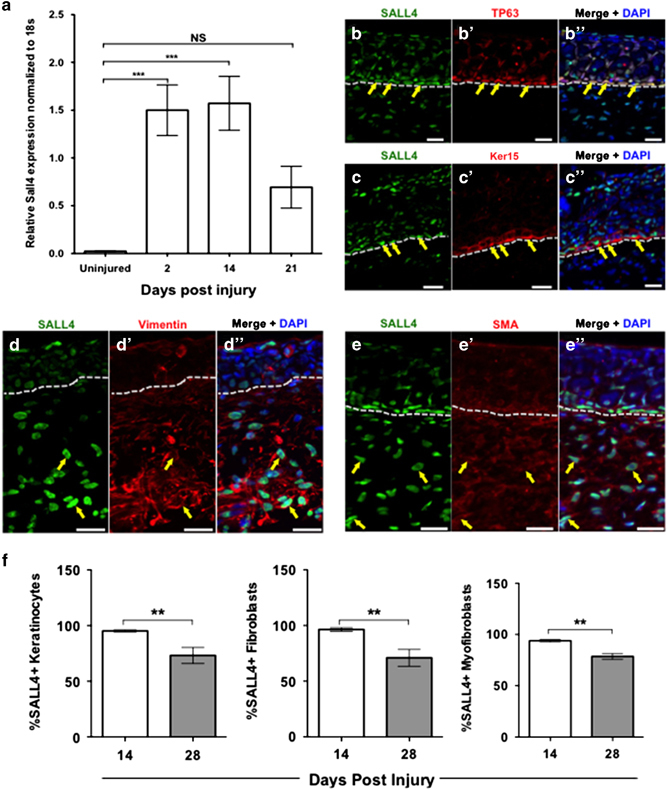
Sall4 expression during wound healing. (**a**) qRT-PCR of Sall4 expression levels during wound healing versus control uninjured tissue. Significance determined by a one-way ANOVA with Dunnett’s multiple comparisons test. *N*=3. Error bars are s.e.m. ***=*P*<0.001. NS=not significant. (**b**–**e″**) Co-Immunofluorescence analysis of SALL4 and TP63 (**b**–**b″**), Keratin 15 (**c**–**c″**), Vimentin (**d**–**d″**) and smooth muscle actin (SMA) (**e**–**e″**) at 28 days post injury. Examples of double positive cells are indicated with yellow arrows. Nuclei are stained with DAPI. Dotted lines represent separation between the epidermis and the dermis. Representative images shown for three replicates of each co-stain. Scale bars = 50 μm. (**f**) The percentage of Sall4+ keratinocytes, dermal fibroblasts (vimentin) and myofibroblasts (smooth muscle actin) are shown within the wound bed at 14 and 28 days post injury. An unpaired two-tailed *t*-test was used to determine significance. Error bars are standard deviation. **=*P*<0.01.

To identify genes whose expression was significantly different in axolotl 2 days post injury compared with uninjured tissue, but unchanged in mammals in the early stages of wound healing, we compared our axolotl wound healing datasets to transcriptional profiles of human wounds during the healing process (GSE28914 and GSE50425).^26^ From the list of 2 days post injury differentially expressed probes in the axolotl microarray data, we identified 571 genes with human homologues, 57 of which were not differentially expressed (Log2-fold change <0.1) at 3 days post injury in the human dataset. Unsupervised hierarchical clustering was applied to the Log2-fold changes for this gene set and changes at each time point are shown in a heatmap (Figure 1). Of these candidates, SALL4 was expressed at particularly high levels in many dermal cells in the axolotl skin during wound healing (Figures 1 and 2; Supplementary Figure 2), thus we focused on SALL4.

To determine the level of conservation between axolotl SALL4 and other species, we cloned the full-length axolotl *Sall4* open reading frame. We identified a 1,146 amino acid open reading frame that has 46% amino acid sequence identity to *H. sapiens* SALL4, 44% identity to *M. musculus* SALL4a and 46% identity to *D. rerio* SALL4 (Supplementary Figure 1). The *A. mexicanium* SALL4 contains seven C2H2 zinc-finger domains that are highly conserved across vertebrate species.

### SALL4 is differentially expressed after skin injury in axolotls

To determine if SALL4 could be having a role in wound healing in the axolotl, we first sought to characterise SALL4 expression in the wound bed. Using quantitative PCR (qPCR), we analysed *Sall4* transcript abundance throughout the course of skin regeneration. Our qPCR data (Figure 2a) supported findings from the axolotl microarray data (Figure 1). Indeed, both analyses showed that *Sall4* transcript abundance increases by two days post injury, remains elevated at 14 days post injury and begins to return to uninjured levels by 21 days post injury (Figure 2a).

A key to understanding the role of SALL4 in axolotl cutaneous wound healing is elucidating which cell types express SALL4. To address this question, we next assayed protein abundance of SALL4 to gain insights into which cells might be expressing SALL4 during the wound healing process in the axolotl. When we used immunofluorescent microscopy to analyse axolotl tissue after wounding, we found that SALL4 protein was localised to cells within the wounded area (Figure 2 and Supplementary Figure 2). Comparatively, no SALL4 was found in the uninjured skin (Supplementary Figure 2, area outside of dashed lines). Co-staining with other cell type-specific antibodies showed that SALL4 was expressed in around 60% basal keratinocytes (TP63 and Keratin 15, Figure 2b,c,f), dermal fibroblasts (vimentin, Figure 2d,f) and myofibroblasts (smooth muscle actin, Figure 2e,f). Interestingly, we also saw that there is a population of cells within the regenerating dermis that are SALL4 positive but do not express markers of other known cells types, this representatives between 4 and 8% of the total cells in the wound bed (Figure 2d and Supplementary Figure 2E). Thus, SALL4 is expressed in multiple cell types in the axolotl cutaneous wound.

### Inhibition of Sall4 leads to early excessive collagen production

**Figure 3:**
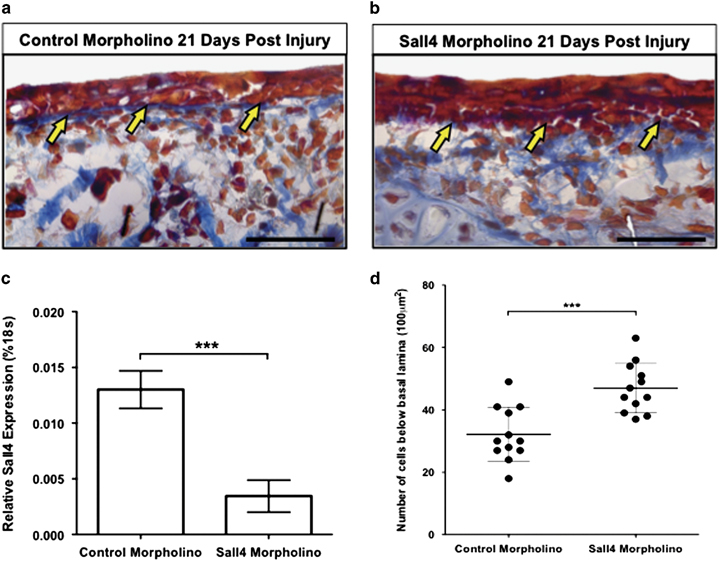
Knockdown of SALL4 leads to excessive collagen deposition during wound healing. Acid fuchsin/ Orange G stain on (**a**) mismatch control morpholino or (**b**) Sall4 morpholino-injected animals at 21 days after injury. Yellow arrows indicate the re-formation of the basal lamina. Representative images shown for two replicates of four animals each. (**c**) Expression of Sall4 relative to 18S at 7 days post injury in tissue that was treated with either mismatch morpholino or Sall4 morpholino. Representative graph of three repeats. Significance determined by an unpaired *t*-test. Error bars are standard deviation (s.d.) ***=*P*<0.0001. (**d**) Analysis of the number of cells in a 100 μm^2^ area below the basal lamina at 21 days post injury after Sall4 knockdown. Replicas=2 with 10 animals total in each condition. Error bars are s.d. ***=*P*<0.001. Scale bar = 20 μm.

We next wanted to test the function of SALL4 during axolotl skin regeneration. Although conventional approaches to deplete *Sall4* have advantages, *Sall4*^*−/−*^ mice are embryonic lethal.^27^ As the same *Sall4-*dependent development is most likely present in the axolotls, we did not produce a body-wide *Sall4*^*−/−*^ axolotl; in addition, we wanted to address the role of SALL4 in regeneration without having previously perturbed development. Instead, to circumvent the role of SALL4 during embryonic development, we used a translation blocking morpholino to specifically deplete SALL4 within the injury site during wound healing. Either the fluorescently tagged morpholino or a mismatched control morpholino were injected and electroporated into the dermal layers during regeneration. qPCR and immunohistochemistry were used to verify that the morpholino successfully reduced both *Sall4* transcript abundance and SALL4 protein during regeneration (Figure 3c; Supplementary Figure 4C,E).

Given previous reports that *Sall4* is necessary for embryonic development and maintenance of stem cells, we expected that regeneration would be delayed on *Sall4* depletion. Instead, we observed that wound closure occurred at a normal rate but later stages of the regeneration process were perturbed, leading to imperfect skin regeneration. To better understand how SALL4 depletion affected the morphology of regenerated skin, we performed acid fuschin-Orange G (AFOG) staining to delineate collagen. We observed that the skin did not return to its normal pre-injury morphology. Histological and immunofluorescent microscopic examination of the regenerating tissue showed that at 21 days after injury, significantly more collagen was present in the SALL4-depleted animals in comparison to the control animals (Figure 3a,b). In addition, there was an increase in the number of cells lying below the basal lamina (Figure 3b,d).

**Figure 4:**
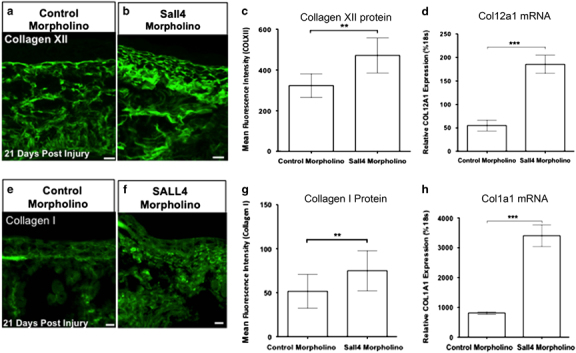
Knockdown of SALL4 leads to increased COL1A and COLXII deposition. (**a**, **b**) Immunofluorescence analysis of Collagen XII 21 days post injury after mismatch morpholino or Sall4 morpholino treatment. (**c**) Quantification of mean fluorescence intensity of collagen XII at 21 days post injury after morpholino treatment. (**d**) Relative expression of COL12A1 to 18S at 14 days post injury after morpholino treatment. (**e**, **f**) Immunofluorescence analysis of Collagen I 21 days post injury, SALL4 depletion also leads to increases Collagen I protein and mRNA levels (**g**, **h**). (**c**–**h**) significance determined by an unpaired *t*-test. Error bars are s.d. unless noted. **=*P*<0.01, ***P<0.001. For 21 days post injury, Replicas=2 with 10 animals total per condition. Scale bar = 50 μm.

Although AFOG can determine if collagen is present it cannot be used to determine the specific type of collagen. From our initial transcriptional profiling approach, we identified 16 collagens that are differentially regulated during skin regeneration in axolotls (Supplementary Figure 3). Therefore, we further examined the type of collagen present in the wound bed of Sall4 knockdown animals using immunohistochemistry and found that this largely consisted of and type I and type XII collagen (Figure 4a,b,e,f). We found that type I and type XII collagen deposition was significantly increased in the wound bed on SALL4 depletion (Figure 4a,b,e,f). In addition to increased collagen I and XII deposition, we noted that collagen was deposited significantly earlier in the SALL4-depleted axolotls than in control axolotls (Supplementary Figures 4A,D and 5A–C). Interestingly, we found that transcription of both type I and type XII collagen was significantly up-regulated in SALL4 knockdown animals (Figure 4d,h). Previous research has shown that in humans after injury collagen is not fully remodelled and generally has a linear pattern.^1,28,29^ We found that as regeneration progressed collagen was remodelled in control axolotls; on SALL4 depletion, there was significantly less remodelled collagen in the wound bed (Figure 4b,c,f,g; Supplementary Figure 4). This finding is reminiscent mammalian scar tissue, where collagen is deposited very early after injury.^1,29–31^

### Sall4 regulates collagen I and XII

Previous literature suggests that type I and type XII collagen are both important components of cutaneous wound remodelling in mammals.^4–6^ Given our data showing an increase in collagen deposition when SALL4 is depleted during wound healing, we questioned if SALL4 might regulate collagen I (*Col1a1)* or collagen XII *(Col12a1)* in the axolotl. Previous studies have reported that SALL4 is able to bind to a Pou5f1-like motif, TTTGCAT in both Mouse and Zebrafish.^14,32^ We identified a single potential SALL4 binding site located within the first intron of *Col1a1* and three separate potential binding sites for SALL4 in *Col12a1*.

**Figure 5:**
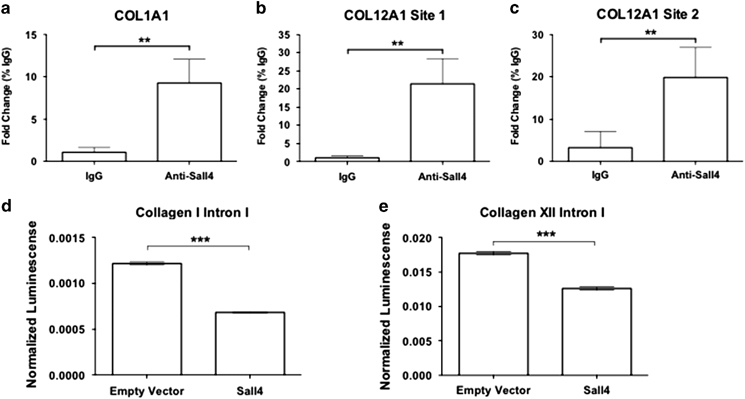
Sall4 binds to collagen promoters and regulates transcription. (**a**–**c**) Chromatin immunoprecipitation with anti-Sall4 followed by qPCR revealed enrichment of collagen I and XII from 14-day injured skin (shown are means of fold change over pull down with IgG). Unpaired *t*-test. Error bars are s.d. **=*P*<0.01. Replicas=2, with each replicate pooling tissue from three animals. (**d**, **e**) A luciferase assay was performed after transfecting HEK293T cells with the pGL3 Enhancer vector containing either (**d**) axolotl collagen I Intron 1 or (**e**) axolotl collagen XII intron 1 together with either a Sall4 over expression construct or empty vector control. After 48 h the cells were lysed and luciferase activity was measured relative to β-galactosidase activity. An unpaired two-tailed *t*-test was used to determine significance. Error bars are s.d. ***=*P*<0.0001.

To determine if SALL4 can bind to regulatory sequences within axolotl collagen genes and regulate their transcription *in vivo*, we harvested axolotl skin at 14 days post injury and performed anti-SALL4 chromatin immunoprecipitation (ChIP) paired with qPCR. First, we found that *Col1a1* regulatory sequences were enriched in the SALL4 immunoprecipitation by 9.27-fold over IgG immunoprecipitation (Figure 5a). We, in addition, found enrichment in two sites of the *Col12a1* regulatory sequences in the SALL4 immunoprecipitation as compared with IgG immunoprecipitation. In *Col12a1* site 1, which contains two SALL4 binding sites, there was a 21.4-fold enrichment of the SALL4 immunoprecipitation as compared with IgG control immunoprecipitation (Figure 5b). Finally, in the *Col12a1* site 2 regulatory sequence, we observed 19.9-fold enrichment in the SALL4 immunoprecipitation as compared with IgG control immunoprecipitation (Figure 5c). This data supports the hypothesis that SALL4 binds to the predicted binding motif in *Col1a1* and *Col12a1*. We confirmed the direct regulation *Col1a1* and *Col12a1* by SALL4 using standard luciferase assays. Cells were transfected with the collagen intron preceding a luciferase reporter, plus or minus axolotl SALL4. Relative luminescence readings were quantified and after addition of Sall4, there was less detectable luminescence when either Col1a1 intron 1 or Col12a1 intron 1 was included upstream of luciferase (Figure 5d,e). This data identifies a previously unknown role of SALL4 in regulating COL1A1 and COL12A1.

### SALL4 is regulated post-transcriptionally by miR-219

**Figure 6:**
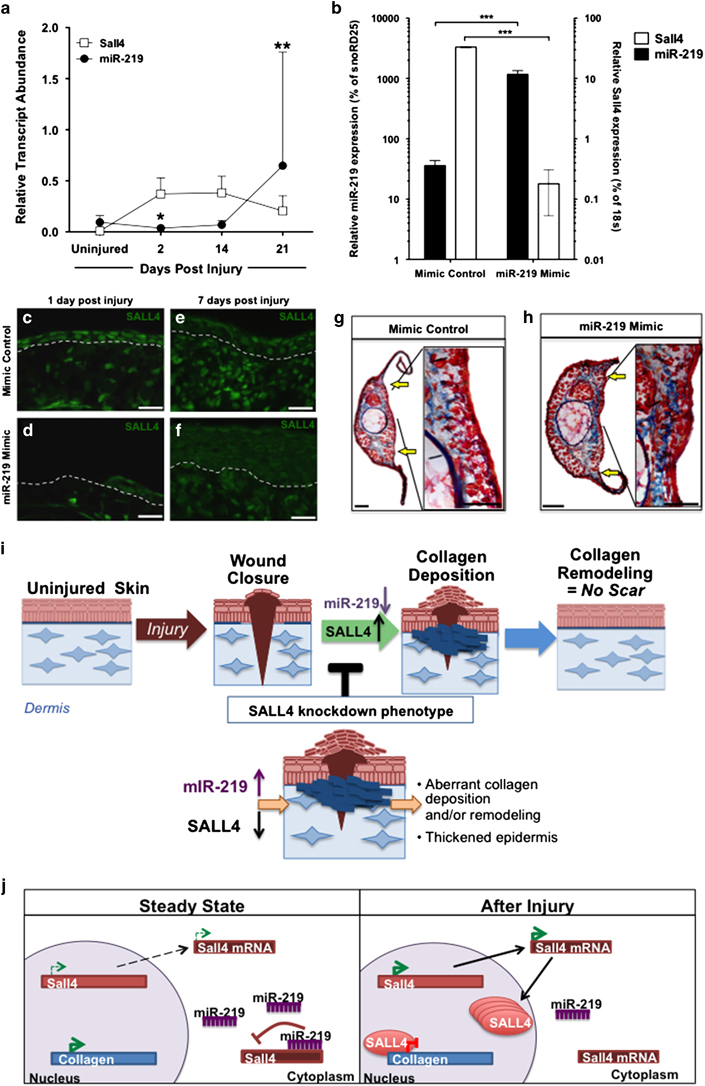
MiR-219 regulates Sall4. (**a**) Log transformed qRT-PCR of Sall4 and miR-219 transcript abundance during wound healing normalised as a per cent of 18S or SnoRD25, respectively. miR-219 levels decrease at 2 days post injury, when SALL4 level increase and at 21 days, miR-219 levels increase, at which time SALL4 levels decrease. Average of samples collected from three different animals. Error bars represent s.d.. Unpaired *t*-test was used to determine significance (**=*P*<0.01, *=*P*<0.05) (**b**) qRT-PCR confirmed that injection of miR-219 mimic into the injury site increased levels of miR-219 transcript and decreased levels of Sall4 message within tissue collected 1 day post injury. *N*=2. Unpaired *t*-test was used to determine significance (***=*P*<0.001) Errors bars=s.d. across four samples, sample are generated from tissue pooled from three animals. (**c**–**f**) Immunofluorescence of SALL4 protein 1 and 7 days post injury in skin samples injected with a non-targeting mimic control or miR-219 mimic. SALL4 protein levels decrease when mature miR-219 mimic is injected into the dermal cells (**d**, **f**). Dashed grey line indicates epidermal/ dermal boarder (Scale bars = 50 μm). (**g**, **h**) Acid fuchsin/ Orange G stain on mimic control or miR-219 mimic-injected animals 7 days after injury. Yellow arrows indicate the injury site. Representative images are from two replicates with 7 or 9 animals total in mimic control or miR-219-injected animals, respectively. Scale bars: **c–f** = 50 μm, **g, h** = 20 μm and inset=10 μm. (**i**) Model illustrating the normal process that leads to scar-free regeneration in axolotls. Deregulation of SALL4 early in regeneration causes a loss of normal repression of collagen due to SALL4 binding to collagen I and 12. This result in early aberrant collagen deposition that does not get remodelled later, ultimately resulting is imperfect skin regeneration. (**j**). A model illustrating the mechanism of SALL4 regulation. In steady state conditions, miR-219 binds to the 3′ UTR of SALL4 and represses its expression. On injury miR-219 repression of SALL4 is released, SALL4 protein then binds to collagen 1 and collagen XII and represses there expression during the early phases of regeneration.

Although we have shown that Sall4 is an important regulator of axolotl skin regeneration, it is still not clear how Sall4 expression is controlled after wounding. We previously demonstrated that Sall4 expression rapidly increases within the wound bed by 2 days post injury and remains elevated until 21 days post injury (Figure 2a). This suggests that *Sall4* transcript abundance is clearly subject to intricate regulation during wound healing. Thus, we wanted to determine what might be controlling SALL4 protein levels during the course of regeneration. MicroRNAs are well-documented to have important roles in regulating genes necessary for regeneration in many models systems.^33–36^ Therefore, we examined the 3′ UTR of axolotl Sall4 and identified one highly conserved seed sequence for microRNA miR-219. Using qRT-PCR we determined the relative levels of miR-219 during axolotl regeneration, looking for a pattern that would be opposing to that of Sall4. MiR-219 showed this pattern, as levels of Sall4 decrease, miR-219 levels increase, as would be expected if the microRNA is binding to the 3′ UTR of the gene and inhibiting its translation (Figure 6a). To determine if Sall4 is a direct target of miR-219, we performed a luciferase assay using the 3′ UTR of the axolotl Sall4 and confirmed that miR-219 can functionally repress Sall4 (Supplementary Figure 6).

To functionally determine if miR-219 levels inversely affect Sall4 levels *in vivo*, a chemically synthesised mature form of miR-219 was injected and electroporated into the axolotl skin during regeneration. Using this approach, combined with qRT-PCR and immunohistochemistry, we show transcription and translation of SALL4 are inhibited by miR-219 mimic in comparison to control mimics (Figure 6b–f). Importantly, we found that depleting Sall4 levels by modulation of miR-219 phenocopied our Sall4 knockdown experiments resulting in excessive collagen deposition (Figure 6g,h), further validating the Sall4 knockdown phenotype observed using Sall4 morpholino. Altogether these data show that dynamic miR-219 expression can regulate Sall4 expression *in vivo* and that perturbation of miR-219 expression leads to excessive collagen deposition, phenocopying the early Sall4 depletion phenotype (Figure 6i,j).

## Discussion

In this study, we have identified a novel role for the transcription factor SALL4 in regulating collagen expression and deposition during scar-free wound healing in axolotl.

SALL4 is a well-known gene, which in mammals has been shown to be important for maintaining stemness during mammalian embryonic development.^12,14,16,18–20,24,37–40^ SALL4 in mammals was identified based on sequence homology to the previously identified *Spalt* genes in Drosophila. In Drosophila *Spalt* is essential for terminal trunk-structure formation in embryogenesis and imaginal disc development in the larval stages.^41–43^

Previous work has identified SALL4 as a gene that is up-regulated during limb development in mouse, and during both development and regeneration of the limb in *Xenopus*.^21,23,44^ In addition, RNA-seq data from regenerating axolotl limb blastemas identified SALL4 as part of a group of genes up-regulated early in regeneration.^45^ It has been postulated that during regeneration SALL4 acts to keep cells in an undifferentiated state in the blastema during early stages of blastema formation and then may have a role in patterning the regenerating limb at later stages.^23,44,45^ This concept would fit very well with the known roles of SALL4 in maintaining cells in an undifferentiated state during development and with its known role in limb patterning.^14,16,17,21,25,32,46,47^

To begin to elucidate the role of SALL4 in skin regeneration, we used transcriptional profiling at different time points following skin injury in the axolotl to identify key genes that are differentially regulated during regeneration. To further narrow down candidate genes, we then compared our dataset to published data from wound healing in humans. This lead to us to focus in on SALL4, given the previous work on this gene, we also expected that it would be involved in maintenance of a stem cell-like state during the early phases of regeneration. Surprisingly, the knockdown phenotype indicated a role in collagen regulation. This result was further corroborated by the identification of SALL4 binding sites within the introns of collagen I and collagen XII genes and verification of this binding using a ChIP assay and data from luciferase assays showing functional regulation of these binding sites by axolotl SALL4 (Figure 5). These results indicate a novel and previously unidentified role for SALL4 during scar-free skin regeneration in axolotl. This data suggests that SALL4 may regulate the timing of collagen expression during regeneration and this may be an important aspect of the whether tissues scar or do not scar after injury. SALL4 is well-documented to have a role in maintenance of the differentiation state. towing to the lack of markers in the axolotl, we could not currently determine if SALL4 depletion also affected the composition of cells in the dermis, as more axolotl-specific markers are developed it will be important and interesting to also answer this question.

Previous research had identified a species of African spiny mice, *Acomys*, which has the ability to heal skin without any scarring.^48^ This paper suggested that one difference in wound healing abilities might lie in the different types of collagen found predominantly in one species versus the other and the timing of ECM deposition. *Acomys* deposit a large amount of collagen III late during wound healing, whereas *Mus* mice have very little collagen III but large amounts of collagen I and this is deposited very early in the wound healing process. More recent papers further examining the differences in regenerative abilities between spiny mice and normal mice have also identified differences in the amounts of collagens present. In *Mus* at least eight collagens are up-regulated very early after injury, the most prominent being collagen XII, whereas in *Acomys* very few collagens were identified.^49,50^ These recent papers again suggest that it is the timing of collagen deposition that may be a key factor in directing the response to injury towards a reparative scarring response versus a scar-free regenerative response, however, these studies do not give any insights into how this is controlled at a molecular level.

In this study, we have identified a novel mechanism of collagen regulation in axolotl by SALL4. Our data suggests that SALL4 binding to collagen I and collagen XII is a key regulatory step that controls the timing of collagen deposition. In the future it will be important to determine how expression of SALL4 after injury is regulated and which co-factors are potentially necessary to determine its interacting partners in different cell types.

The open question remains as to why mammals do not up-regulate SALL4 after injury. We have examined the human collagen genes and found there are no SALL4 binding sites in COL1A1, but there are seven potential SALL4 binding sites with intron 1 of COL12A1. However, the SALL4 binding motifs identified so far have not been extensively verified, and in addition they are highly homologous to the known Oct4 motif, so further analysis will be needed to definitively determine if the human collagen I genes can be regulated by SALL4. To further dissect the mechanism by which SALL4 is regulated after injury in axolotl, we also examined the 3′ UTR of the gene for potential microRNA seed sequences. We identified a microRNA; miR-219, and have shown that it has a key active role in regulating the expression of SALL4 during wound healing. In addition, we have bioinformatically analysed the 3′ UTR of the human SALL4 gene and found that it does contain a miR-219 binding site. It will be interesting in the future to determine if the timing of collagen deposition can be regulated in the same manner in humans as it is in axolotl and if this will suffice to alter the timing of collagen deposition after injury.

Collagen deposition is only one part of the pathway to scarring. Another key difference between axolotls and mammals is their ability to remodel collagen after deposition to return the skin to its original morphology. A previous study, performed chromatin immunoprecipitation coupled to microarray hybridisation (ChIP-on-chip) in W4 mouse ES cells to determine which genes could be bound by SALL4.^14,18,40,47^ This ChIP-chip indicates that SALL4 could potentially bind to and regulate several collagen-remodelling genes, such as LOXL2 and several MMPs. It will be essential in the future to investigate how species that heal scar-free can in fact remodel collagen.

In summary, the data within provide novel mechanistic insights into how the axolotl regulates collagen during scar-free wound healing and it identifies a previously unknown role for SALL4 in regulating collagen transcription. In the future, it will be interesting to determine how conserved this new role for SALL4 is across different species.

## Materials and methods

### Axolotls

All axolotls used in these experiments were housed at the University of Minnesota in accordance with IACUAC protocol number 1411–32049A. Adult and larval (2–5 cm) were used for these experiments. Before *in vivo* experiments, animals were anaesthetised using 0.1% *p*-amino benzocaine (Sigma, St Louis, MO, USA). Skin biopsy punches were performed using a sterile 2 mm disposable biopsy punch with plunger (Miltex, York, PA, USA). At desired time points, tissue was collected using a sterile scalpel. The collected tissue was either immediately placed into TRIzol (Ambion, Waltham, MA, USA) or 4% paraformaldehyde (Sigma).

### Microarray

Uninjured, 2, 14 and 21-day post injury samples were generated from three separate adult male animals by extracting Total RNA using the TRIzol protocol (Invitrogen, Waltham, MA, USA). Full thickness 2 mm biopsy samples were taken from the skin along the body of adult axolotls that were 2 years old and measuring between 12 and 15 inches from tip of head to tip of the tail. All probe preparation, hybridisation and quality control was carried out by the DNA Microarray Core Facility at the Max Planck Institute CBG, Dresden. Custom Affymetrix GeneChimb Amby002 arrays were used for genome wide expression analysis. This array has ~20,000 unique probe sets. Probe annotations are from Sal-Site (www.ambystoma.org). Array quality and differential gene expression was assessed using standard microarray techniques in R/Bioconductor using custom scripts. All arrays were deemed of high quality and were included in all following analysis. Background correction, normalisation and expression summaries were obtained using the robust multi-array average (RMA) algorithm. Differential gene expression was examined using the limma R/Bioconductor package (https://www.bioconductor.org); *P* values were adjusted for multiple comparisons using the Benjamini and Hochberg method. The final list of differentially regulated genes was further analyses for pathway interaction using the Ingenuity Pathway Analysis software. This data has been submitted and its GEO accession number is GSE79299.

### Comparison of axolotl array data to publically available human array data

This custom array contains probes designed against 20,031 axolotl contigs. Of these contigs, 14,976 contained significant homology to human transcripts to allow for cross species comparison. Fluorescence intensities from these arrays were background corrected and normalised using the robust multi-array average (rma) within the Bioconductor affy package.^51^ A linear regression model was fit to the data using the limma software.^52^ Previously published data in human was downloaded and processed as above from Gene Expression Omnibus accession numbers GSE50425 and GSE28914.^26^ Axolotl transcripts that were twofold differentially expressed between the uninjured and 2 day post injury time point and had an adjusted *P* value below 0.05 were cross-referenced to the human transcripts that were differentially expressed at 3 days post injury in the published data. Log2-fold changes in the axolotl for all three time points compared with the uninjured control was plotted for this subset of genes using the pheatmap package in R. A detailed informatics workflow can be found at https://github.com/micahgearhart/sall4.

### Quantitative PCR

Total RNA samples were extracted using TRIzol. All samples were treated with DNase (NEB) for 30 min at 37 °C to remove any DNA contamination. DNase was inactivated by addition of 25 mmol/l EDTA and incubation at 65 °C for 15 min. cDNA was produced by using miScript II RT Kit (Qiagen, Hilden, Germany) per manufacturer’s instructions. cDNA samples were diluted 1:2 before qPCR. The qRT-PCR was carried out using miScript SYBR Green PCR kit (Qiagen) per manufactures protocol. Either Qiagen designed primers compatible with miScript kit were purchased to quantify conserved microRNAs, and custom designed primers were made by IDT to amplify axolotl mRNAs. Custom axolotl primers used:

18S_F: CGGCTTAATTTGACTCAACACG

18S_R: TTAGCATGCCAGAGTCTCGTTC

Sall4_F: AATCCCTCGCAAGCCC

Sall4_R: CCAGCTATGAGGGGAACATT

Collagen I_F: TCCCAAAACATCACCTACCAC

Collagen I_R: AGCTCTGATCTCAATCTCGTTG

Collagen XII_F: TCAGCGTGAATTCTGTGTAGG

Collagen XII_R: CTTCGACGTGTCTCCTGAAAG

### Immunohistochemistry

Tissue was fixed with 4% paraformaldehyde and embedded in Tissue-Tek O.C.T. (Sakura, Alphen aan den Rijn, Netherlands) for sectioning. 20 μm sections were stained using standard immunofluorescence techniques. All sections underwent epitope retrieval by incubation in PBS at 70 °C for 20 min. Sections were permeablized by washing three times for 10 min with 0.1% Triton X-100 (Sigma) in PBS and blocked with 2% Goat serum and 2% BSA Fraction V (Sigma) in 0.1% Trition X-100 PBS for one hour at room temperature. The following primary antibodies were used: rabbit anti-SALL4 (1:250; 8459S Cell Signaling Technology, Danvers, MA, USA), Mouse anti-p63 (1:200; MAB4135 Millipore, Billerica, MA, USA), mouse anti-cytokeratin 15 (1:200, ab80522 Abcam, Cambridge, UK), mouse anti-Vimentin 40EC (1:100, Developmental Studies Hybridoma Bank, Iowa City, IA, USA), mouse anti- alpha smooth muscle actin (1:500, A5228 Sigma), mouse anti-collagen XII MT2-s (1:100, Developmental Studies Hybridoma Bank), anti-mouse collagen I (1:500, C2456 Sigma. Antigen retrieval of 5 μg/ml Proteinase K digest in TE for 10 min at 37 °C). Primary antibodies were diluted with blocking buffer. After four 5-min washes with PBS plus 0.1% Tween 20 (Sigma), slides were incubated with AF488-, AF568- or AF647-conjugated goat anti-rabbit or mouse secondary antibodies (1:200) in blocking buffer. Nuclei were stained with DAPI. Slides were washed four more times for 10 min with 0.1% Tween 20 PBS and mounted with mounting media and coverslips. Slides were analysed using Leica DMI 6000 B epifluorescent microscope driven by Leica LAS AF software (Leica, Wetzlar, Germany).

### Image analysis

The open source software Fiji was used to process all images acquired on the Leica microscope. Fluorescence intensity was quantified using Matlab from the raw images acquired from sections stained with antibodies against SALL4 and ColXII. A custom MATLAB (Mathworks, Version 8.2, Natick, MA, USA) script was used to analyse the images, Figure 4 and Supplementary Figures 3 and 4. Matlab code is available by request. Each image was displayed using all gathered colour channels to simplify identification of sample containing regions of the image. Sub-sections of the images were manually selected to include only portions of the images containing the sample. This avoids the background regions altering the fluorescence intensity statistics. The intensity statistics were exported; one set of statistics for each image, and subsequently visualised using Excel.

### Microinjection and electroporation

Microinjections of miR-219 mimics (Qiagen), morpholinos (Gene Tools, Philomath, OR, USA) or controls were carried out as previously described in Erickson and Echeverri.^53^ Briefly, miR-219 mimic or control was diluted to a final concentration of 10 μM in PBS plus Fast Green. Morpholinos were diluted to a final concentration of 1 mmol/l in sterile DI water plus Fast Green. World Precision Instruments pressure injector was used to inject the solutions directly into the wound bed every other day until collection. Post injection, axolotls are placed in PBS and electroporated with five pulses of 50 V, 50 ms each. When tissue samples were harvested at 7 or 21 days post injury multiple injection of the morpholino, mimics or controls were given over that time period.

Am Sall4 Morpholino: GACCTGGAAAAAACCCAGTCATTGC

Control Morpholino: GAACTGCAAAAAAACAGTAATTCC

### Acid fuchsin/ Orange G staining

Acid fuchsin/ Orange G staining on fixed tissue was carried out as previously described in Diaz *et al.*^54^ 2014. Briefly, tissue samples were collected and fixed in 4% paraformaldehyde (Sigma). Tissue was then embedded using Tissue-Tek (Sakura) and 20 μm cross sections were taken and slides were post fixed with Bouin’s Solution overnight. Sections were washed with distilled water and then stained with successive 5-min incubations in 1% phosphomolybdic acid (Sigma), AFOG solution and 0.5% acetic acid. Slides were washed with distilled water for 5 min before and after incubation with AFOG solution. After staining, slides were successively dehydrated by 2 min incubations in 96% and 100% ethanol, followed by xylene incubation for 5 min before mounting with 80% glycerol (Sigma). Images were captured using an Olympus BX40 inverted microscope with Leica EC 3 camera (Leica) and Leica Acquire software (Leica).

### Chromatin immunoprecipitation

Chromatin immunoprecipitation was carried out using SimpleChIP Plus Enzymatic Chromatin IP Kit (Magnetic beads) (Cell Signaling Technologies #9005) following the manufacturers protocol. Briefly, 0.1 mg of tissue was collected 14 days post injury. Tissue was cross-linked with 1.5% formaldehyde at room temperature for 20 min. Cross-linking was stopped with glycine addition and incubation for 5 min at room temperature. Tissue was washed and dissociated using a pestle motor mixer (Argos Technologies, Elgin, IL, USA) and pestle. Samples were then incubated with Micrococcal Nuclease for 20 min at 37 °C and inverted every 3–5 min. The digestion was stopped by addition of EDTA. Samples were washed and then sonicated on ice with six 15-s pulses with 45 s of rest. The cross-linked chromatin preparation was then isolated and analysed to ensure chromatin was the appropriate size and concentration. A 2% input of each sample was set aside. The remaining cross-linked chromatin preparation was then split into three tubes and incubated with either the positive control, 10 μl Histone H3 (D2B12) XP Rabbit mAb (Cell Signaling Technologies), the negative control, 1 μl Normal Rabbit IgG (Cell Signaling Technologies) or 10 μl anti-SALL4 (8459S Cell Signaling Technology) and incubated overnight with rotation at 4 °C. Each sample was then incubated with ChIP-Grade Protein G Magnetic Beads for 2 h at 4 °C with rotation. The Protein G Magnetic beads were pelleted and washed by placing tubes in a magnetic separation rack. Chromatin was eluted from the magnetic beads by incubation at 65 °C for 30 min with intermittent vortexing. The magnetic pellets were then removed by incubating on a magnetic separation rack. The supernatant containing chromatin was then collected and cross-links were reversed by Proteinase K digestion at 65 °C overnight. DNA was then purified using DNA purification spin columns. DNA was eluted from the columns with 50 μl of DI water. PCR and qPCR were then performed to evaluate SALL4 binding using the following primers:

Collagen I For: GTTTCTTTTCACTGTGCCCG

Collagen I Rev: CGAGAGTTCCCATGCCATAG

Collagen XII Site 1 For: GAGCACTGTCCTCTTAGACAAG

Collagen XII Site 1 Rev: CCACAAAGCCACCCAGTAG

Collagen XII Site 2 For: CTGGCTAATAATCACAATCCTGTCT

Collagen XII Site 2 Rev: TAGGTTTTTGTAAGTGGTCCAGTG

### Cloning and plasmids

The full-length axolotl Sall4 was cloned out of RNA isolated from tissue collected at 14 days post injury. 5′ and 3′ RACE was performed using Smart RACE kit (Clontech, Mountain View, CA, USA) and following manufacturer’s instructions and the following primers:

SALL4 Rev GSP1: CCTTAAGATTCCCTTTCG

SALL4 Rev NGSP1: GCAGCGCACTATCATTCCCAAAGAC

SALL4 Rev GSP1_2: GATTACGACCAAGCTTCAGAGAGCTGCTGAGACAGGTGAGCCC

SALL4 Rev NGSP1_2: GATTACGACCAAGCTTCGGATGGGTGAAGAACGTGAGGCGCC

SALL4 Rev GSP1_3: GATTACGACCAAGCTTCCCGCAACTCCGCATGCAGCCACAGC

SALL4 Rev NGSP1_3: GATTACGACCAAGCTTAGCAGTACCAGCAACAAGGAGCAACTTGTC

SALL4 3'RACE GSP2: GATTACGACCAAGCTTGCTCCAGCGGCTTGTTGAGAACATTGACCG

SALL4 3'RACE NGSP2: GATTACGACCAAGCTTCTCTGTGGCCGTGCTTTCTCAACCAAGGG

Axolotl Sall4 3′ UTR was cloned out of RNA extracted from a 14-day post injury skin wound using the primers indicated below. After restriction digest with SpeI and NotI (NEB) of both the PCR fragment and the pMIR-Report Luciferase vector (Life Technologies, Waltham, MA, USA), the fragments were ligated overnight at 4 °C with T4 Ligase (Promega, Madison, WI, USA). Ligated plasmids were transformed into DH5α *E. coli* (Invitrogen). Plasmid DNA was then isolated using Qiagen’s Midiprep kit per manufacturer’s instructions.

AxSall4 3′ UTR SpeI_F: CTGACTAGTCATCGCTGTCAGTTGAGG

AxSall4 3′U TR NotI_R: GCAGCGGCCGCGTGGTATCAACGCAGAGTAC

The full-length axolotl SALL4 was deposited to GenBank, accession number: KX035097.

Primers:

For- GGGGACAAGTTTGTACAAAAAAGCAGGCTgccaccatggactacaaagacg

Rev- GGGGACCACTTTGTACAAGAAAGCTGGGTctagatcacaccttcctcttct

COLL1A, COLXII and SALL4 constructs.

Intron 1 of axolotl Col1a1 and intron 1 of Col12a1 were PCR amplified from genomic DNA using the primers listed below. After restriction digest with NheI-HF and XhoI (NEB) of both the PCR product and pGL3 Enhancer vector (Promega), fragments were ligated at 4 °C overnight with T4 ligase (NEB). DH5α *E. coli* were transformed with ligation reactions and selected for using ampicillin. Plasmid DNA was then isolated using Qiagen’s Midiprep kit per manufacturer’s instructions.

ColI Intron For pGL3 NheI: CATG**GCTAGC**CAAGAAGACGGTAAGTAGCAC

ColI Intron full Rev: CATG**CTCGAG**TCGCACACGCAGATCGTG

Col XII Intron For pGL3 NheI: CATG**GCTAGC**CAAGCAACCAGGGGAGGA

ColXII intron full Rev: CATG**CTCGAG**CCACAGAGGCGGCTCCAGATTGTG

Full-length axolotl Sall4 was cloned out of RNA extracted from a 14-day post injury skin wound using the primers listed below using Qiagen One Step RT-PCR kit following manufacturer’s protocol. A restriction digest with NheI and BamHI (NEB) was performed on both the PCR fragment and pcDNA3.1 MCS-BirA(R118G)-HA. pcDNA3.1 MCS-BirA(R118G)-HA was a gift from Kyle Roux (Addgene plasmid # 36047). Digested PCR product and vector were then ligated using T4 DNA ligase (NEB) overnight at 4 °C. DH10β *E. coli* were then transformed with the ligation reaction and selected with kanamycin. Plasmid DNA was purified using Qiagen’s Midiprep kit per manufacturer’s instructions.

AxSall4 HA BioID NheI For: CATGGCTAGCCCACCATGAGCCCAGAGCCTGCATC

AxSall4 HA BioID BamHI Rev: CATGGGATCCACTGACAGCGATGTTACTTTCCTCCA

### Site-directed mutagenesis

The miR-219 seed sequence was mutated by performing PCR using FastStart Taq DNA polymerase dNTPack (Roche) on the pMIR-Report luciferase-AxSall4 3′ UTR plasmid with the following primers:

AxSall43′ UTR SDM For1: CTGCGCACTAGTCATCGCTGTCAGTTGAGG

AxSall43′UTR SDM Rev1: AAGCATAGTCA**TGGTACC**CCTCTGGCCAAC

AxSall43′UTR MSDM For2: GTTGGCCAGAGG**GGTACCA**TGACTATGCTT AxSall43′ UTR MSDM Rev2: GCTAGCGGCCGCGTGGTATCAAC

The bold underlined bases are non-complementary sequence located where the miR-219 seed sequence is located. The two fragments were purified and combined in a PCR reaction with AxSall43′ UTR SDM For1 primer and AxSall43′ UTR MSDM Rev2 primer. This gave one PCR fragment with the mutated miR-219 seed sequence. Both the fragment and pMIR-Report Luciferase vector (Life Technologies), were digested with SpeI and NotI (NEB) and the fragments were ligated using T4 Ligase (Promega) overnight at 4 °C. Ligation reactions were then transformed into DH5α *E. coli* (Invitrogen). Plasmid DNA was then isolated using a Midiprep kit (Qiagen).

### Luciferase assay

#### Col1a1 and col12a1 luciferase assays

To determine if Sall4 could functionally regulate transcription of Col1a1 or Col12a1, HEK293T cells were plated at a density of 2×10^4^ cells per well in a 96-well plate coated with poly-D-lysine and allowed to adhere overnight. HEK293T cells were then co-transfected with either 112.5 ng per well of pGL3 Enhancer Col1a1 Intron 1, 37.5 ng per well pMIR-Report beta gal vector (Life Technologies) and 50 ng per well pcDNA3.1 MCS-BirA(R118G)-HA (with or without axolotl Sall4 insert) or 125 ng per well of pGL3 Enhancer Col12a1 Intron 1, 25 ng per well pMIR-Report beta gal vector (Life Technologies) and 50 ng per well pcDNA3.1 MCS-BirA(R118G)-HA (with or without axolotl Sall4 insert) using Lipofectamine 3000 (Invitrogen). Cells were incubated for 48 h and luminescence was detected using the Dual Light System (Ambion) following the manufacturer’s protocol.

#### miR-219 and SALL4 luciferase assay

To determine if Sall4 is a target of miR-219, HEK 293 cells were plated in a 96-well cell culture plate at a density of 1.2×10^4^ cells per well and were allowed to adhere overnight in D-MEM (Gibco, Waltham, MA, USA) with 10% FBS (Thermo Scientific, Waltham, MA, USA). HEK cells were then co-transfected with 135 ng per well pMIR-Report Vector (with or without Sall4 3′ UTR insert or seed sequence mutagenized SAll4 3′ UTR insert) and 45 ng per well pMIR-Report beta gal vector (Life Technologies) with 100 nmol/l miR-219 mimic or mimic control using Lipofectamine 2000 (Invitrogen). Cells were incubated for 48 h and luminescence was detected using the Dual Light System (Ambion) using the manufacturer’s protocol.

### Statistical analyses

All results are presented as mean±s.e.m. unless otherwise stated. Analyses were performed using Microsoft Excel or GraphPad Prism. Dataset means were compared using ANOVA for three or more tests. When two groups were compared a Students *t*-test was used. Differences between groups was considered significant at three different levels (*P* values of <0.05, <0.01 and <0.001) and are indicated in the figure legends.
