# Supplementary material for: A novel role for SALL4 during scar-free wound healing in axolotl
Source: NPJ Regen Med. 2016 Dec 8;1:16016–. doi: 10.1038/npjregenmed.2016.16 (PMC5612448; doi:10.1038/npjregenmed.2016.16)
Supplement: Supplementary Figure 1 [file npjregenmed201616-s1.pdf]

```
logo
X tropicalis  MSRRKQPNPQHLNSEEQL........................LDAS.AEQKDCPSNTDGERAVKRCRMEETHICEKCCAEFFELSDLLEHKKSCTKNPPVLIMNDSEGAM   85
G gallus      MSRRKQAKPQHINSEEQP........................PDAXXGSPRDVPGDGDGEMSSKRCRTEETKICEKCCAEFFDLSEFLEHKKNCTKNPPVLIMNDSEGAV   86
A mexicanum   ............MSPEPA........................SGNLAAELNGCRSDGDGKMSVKRCRMEETHVCEKCSAEFFDLSKFIQHKKSCPKTTPVLIMNDRKGVL   74
D rerio       MSRRKQSKPQHINSDDPA........................STEN.GILHNSQSEEDGS.DAKRRRSEETRVCEKCCAEFFDEAEFLEHERNCTKSQQVVIMKDGDGSE   84
H sapiens     MSRRKQAKPQHINSEEDQGEQQPQQQTPEFADAAPAAPAAGELGAPVNHPGNDEVASEDEATVKRLRREETHVCEKCCAEFFSISEFLEHKKNCTKNPPVLIMNDSEGPV  110
M musculus    MSRRKQAKPQHINWEEGQGEQPQQLPSPDLAEALAAE....EPGAPVNSPGNCDEASEDSIPVKRPRREDTHICNKCCAEFFSLSEFMEHKKSCTKTPPVLIMNDSEGPV  106
consensus     ****** *********                            *  *         **   *!! !  !*!**!*!!*!!!!******!** !*!  **!*!!*!**! *

logo
X tropicalis  PHDGYAEDSPEGAEAEPLEVPTVDNVQPKSSSVSPEKMEEKVDSA.........KKTVPQSTPKTNGLGYVSKTKLSNTNVTLQTINSTKVAVNQHAADGVATSQSNPN.  185
G gallus      PPEGFSEAAVGSFSSDRGDSRAHKDTQAKGCTGSAEKREGKIDAESVGGM...YLKIEPPAAPVTPGLSYLPKSKVPNTNVTLQTIRGTKVAVNQRTTDAIASSAASFN.  192
A mexicanum   RTEGFPESSP...VRNGMMSPNAHDVQPESSNGYVDKIKEETSPTPIASVGSPYQKTPSPSIPTPPAVSYLP..KVPNTNVTLQTIHGTKVAVNQHTADTVSPQSVGFN.  178
D rerio       VPPEFSQRSPGDVLSDP..CDQSTNSYSKHGAESDEMMEGEFMLNQ........DNPSNHDQEVSGSPGYVASSKLQDSNVTLESMAATKVAVTQHSSNSSSQKSPPPQP  184
H sapiens     PSEDFS....GAVLSHQPTSPGSKDCHRENGG.SSEDMKEKPDAES.....VVYLKTETALPPTPQDISYLAKGKVANTNVTLQALRGTKVAVNQRSADALPAPVPGAN.  209
M musculus    PSEDFS....RAALSHQLGSPSNKDSLQENGS.SSGDLK.KLGTDS.....ILYLKTEATQPSTPQDISYLPKGKVANTNVTLQALRGTKVAVNQRGAEAPMAPMPAAQ.  204
consensus     * *  **       **    **   *        * * *  *            *  **     *   **!*  *  !*  **!!!!***  *!!!!!!*!  *****       *

logo
X tropicalis  ......AIPMILEQLVCLQQQQLQQIQLTEQIRIQIAMMAPNSLHPSIAAATDPLKALGAHLS......QQLSAAVALIGQKAGTQSLSLESLKQSKLPHSTVAMPTAGT  283
G gallus      ......AIPMILEQLVCLQQQQLQQIQLTEQIRIQIAMMAPHALHPSIAAAADPLKALGAHMS......QQLSAAVALIGQKAGSQSLSLESLKQGKLPHANTGVAAAGS  290
A mexicanum   ......AIPLILEQLVCLQQQQLQQIQLTEQIRIQIAMMAPHVLHPSVASASDPLKALGAHLS......QQLSAAVALIGQKACHQSLSLESFMQGKIAPPNMVMS.AGA  275
D rerio       HQDTLLAIPMILEQLVSLQQQQLQQIQLTEQIRIQVAMMAPPSLHAAVGAVMDPLKALGAHLS......QQLSAAAALIGKRTGSQSLSLEALKQAKLPQSATIPT...S  285
H sapiens     ......SIPWVLEQILCLQQQQLQQIQLTEQIRIQVNMWASHALHSSGAG.ADTLKTLGSHMS......QQVSAAVALLSQKAGSQGLSLDALKQAKLPHAN.IPSATSS  305
M musculus    ......GIPWVLEQILCLQQQQLQQIQLTEQIRVQVNMWAAHALHSGVAG.ADTLKALSSHVSQQVSVSQQVSAAVALLSQKASNPALSLDALKQAKLPHAS.VPSAASP  306
consensus           *!!**!!!***!!!!!!!!!!!!!!!!*!**!*!**  !!  ****   !*!!*!**!*!      !!*!!!*!!*******!**!!!*  **!*!***   *  *  * *

logo
X tropicalis  VPLALTTSL.LKQEPSLGLSTAVGRFPNPALPHSPGTVIFQNPINAL....DPSKKLKGKFPSVTTPE....TKPGGEDQFFRHKCKFCGKVFGNDSALQIHLRSHTGER  384
G gallus      LAAGLSSPFPLKPEASRTFAGSISRFPNPLLPQSSHSVIFQNPLSAVSSVIDSSKRVKGKPPNICVSE....SKSSAEEPFFKHKCKFCGKVFGNDSALQIHLRSHTGER  396
A mexicanum   RPIGLPSSH.FKPEANRMIHNSVARFPNPSQAQPPKSVISQNPALSLASTMDSSK..KGKPPNIGVSE....NRPTPDESFFKHKCKFCGKVFGNDSALQIHLRSHTGER  378
D rerio       LPGGLGSIP.LKPD.LKGLPDLASRLP.ALLPQSPGVIGLQSPFNNLATSMDPSKKAKTKGPVVSEP.....PKNVSVEHSFKHKCKFCGKTFGNDSALQIHLRSHTGER  387
H sapiens     LSPGLAPFT.LKPDGTRVLPNVMSRLPSALLPQAPGSVLFQSPFSTVA..LDTSKKGKGKPPNISA....VDVKPKDEAALYKHKCKYCSKVFGTDSSLQIHLRSHTGER  408
M musculus    LSSGLTSFT.LKPDGTRVLPNFVSRLPSALLPQTPGSVLLQSPFSAVT..LDQSKKGKGKPQNLSASASVLDVKAKDEVVLGKHKCRYCPKVFGTDSSLQIHLRSHTGER  413
consensus     *   *! *   *!***** *    **! !   *********  !  !   *     *! !!* !*!*****       *    **   **!!!**!*!*!*!!!*!!*!!!!!!!!!!!!

logo
X tropicalis  PYKCNICGNRFTTKGNLKVHFQRHKDKYPHIKMNPYPVPEHLDNVPTTSGIPYGMSVP..LDESNVIVDTKSGVAGLPN......ATNLSSLTET...VLGAFPLNMQSR  483
G gallus      PYKCNICGNRFTTKGNLKVHFQRHKDKYPHIKMNPYPVPEHLDNVPTSSGIPYGMSVP..LDESNLIVDSKPLLTTLPTSAASSAPPNAPSLAGTK..EPAAFSTELPSR  502
A mexicanum   PFKCNICGSRFTTKGNLKVHFQRHRDKYPHIRMNPYPVPEHLDNVPTPSGIPYGMSVP..LDESNVLADIKPVLSSLPIG..LNLSQNVPLIAGIKDSNGGSLPTNVQSR  484
D rerio       PFKCNICGNRFTTKGNLKVHFQRHKEKYPHIKMNPHPVPEHLDNMPSNNGIPYGMSVP..MEE.NGFSETKPVLGVPTTGPPAGPHPPVLQAFKPSFDIPAAGDPYSQRP  494
H sapiens     PFVCSVCGHRFTTKGNLKVHFHRH....PQVKANPQLFAEFQDKVAAGNGIPYALSVPDPIDEPSLSLDSKPVLVT..TSVG..LPQNLSSGTNPKDLTGGSLPGDLQPG  510
M musculus    PYVCPICGHRFTTKGNLKVHLQRH....PEVKANPQLLAEFQDKGAVSAASHYALPVPVPADESSLSVDAEPVPVTGTPSLG..LPQKLTSGPNSRDLMGGSLPNDMQPG  517
consensus     !**!**!! !!!!!!!!!!!**!!****!****!!  ***!**!****  ***!***!!   **!*** ****** *              ** *    *    *  *  **
```

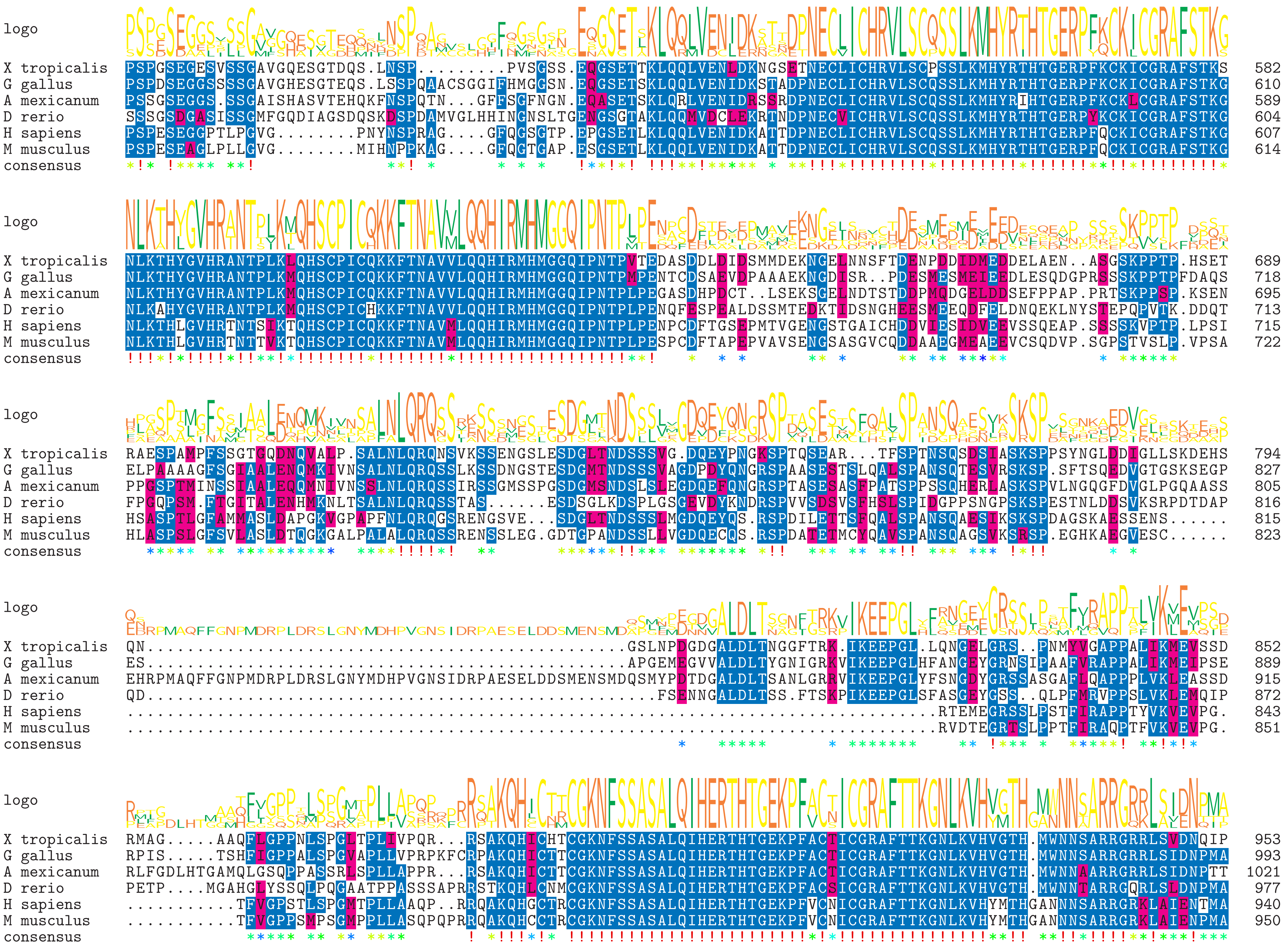
logo
X tropicalis PSPGSEGESVSSGAVGQESGTDQS.LNSP.........PVSGSS.EQGSETTKLQQLVENLDKNGSETNECLICHRVLSCPSSLKMHYRTHTGERPFKCKICGRAFSTKS 582
G gallus PSPDSEGGSSSSGAVGHESGTEQS.LSSPQAACSGGIFHMGGSN.EQGSETSKLQQLVENIDKSTADPNECLICHRVLSCQSSLKMHYRTHTGERPFKCKICGRAFSTKG 610
A mexicanum PSSGSEGGS.SSGAISHASVTEHQKFNSPQTN...GFFSGFNGN.EQASETSKLQRLVENIDRSSRDPNECLICHRVLSCQSSLKMHYRIHTGERPFKCKLCGRAFSTKG 589
D rerio SSSGSDGASISSGMFGQDIAGSDQSKDSPDAMVGLHHINGNSLTGENGSGTAKLQQMVDCLEKRTNDPNECVICHRVLSCQSSLKMHYRTHTGERPYKCKICGRAFSTKG 604
H sapiens PSPESEGGPTLPGVG........PNYNSPRAG....GFQGSGTP.EPGSETLKLQQLVENIDKATTDPNECLICHRVLSCQSSLKMHYRTHTGERPFQCKICGRAFSTKG 607
M musculus PSPESEAGLPLLGVG........MIHNPPKAG....GFQGTGAP.ESGSETLKLQQLVENIDKATTDPNECLICHRVLSCQSSLKMHYRTHTGERPFQCKICGRAFSTKG 614
consensus
logo
X tropicalis NLKTHYGVHRANTPLKLQHSCPICQKKFTNAVVLQQHIRMHMGGQIPNTPVTEDASDDLDIDSMMDEKNGELNNSFTDENPDDIDMEDDELAEN..ASGSKPPTP.HSET 689
G gallus NLKTHYGVHRANTPLKMQHSCPICQKKFTNAVVLQQHIRMHMGGQIPNTPMPENTCDSAEVDPAAAEKNGDISR..PDESMESMEIEEDLESQDGPRSSSKPPTPFDAQS 718
A mexicanum NLKTHYGVHRANTPLKMQHSCPICQKKFTNAVVLQQHIRMHMGGQIPNTPLPEGASDHPDCT..LSEKSGELNDTSTDDPMQDGELDDSEFPPAP.PRTSKPPSP.KSEN 695
D rerio NLKAHYGVHRANTPLKMQHSCPICHKKFTNAVVLQQHIRMHMGGQIPNTPLPENQFESPEALDSSMTEDKTIDSNGHEESMEEQDFELDNQEKLNYSTEPQPVTK.DDQT 713
H sapiens NLKTHLGVHRTNTSIKTQHSCPICQKKFTNAVMLQQHIRMHMGGQIPNTPLPENPCDFTGSEPMTVGENGSTGAICHDDVIESIDVEEVSSQEAP.SSSSKVPTP.LPSI 715
M musculus NLKTHLGVHRTNTTVKTQHSCPICQKKFTNAVMLQQHIRMHMGGQIPNTPLPESPCDFTAPEPVAVSENGSASGVCQDDAAEGMEAEEVCSQDVP.SGPSTVSLP.VPSA 722
consensus
logo
X tropicalis RAESPAMPFSSGTGQDNQVALP.SALNLQRQNSVKSSENGSLESDGLTNDSSSVG.DQEYPNGKSPTQSEAR...TFSPTNSQSDSIASKSPPSYNGLDDIGLLSKDEHS 794
G gallus ELPAAAAGFSGIAALENQMKIVNSALNLQRQSSLKSSDNGSTESDGMTNDSSSVAGDPDYQNGRSPAASESTSLQALSPANSQTESVRSKSP.SFTSQEDVGTGSKSEGP 827
A mexicanum PPGSPTMINSSIAALEQQMNIVNSSLNLQRQSSIRSSGMSSPGSDGMSNDSLSLEGDQEFQNGRSPTASESASFPATSPPSSQHERLASKSPVLNGQGFDVGLPGQAASS 805
D rerio FPGQPSM.FTGITALENHMKNLTSALNLQRQSSTAS......ESDSGLKDSPLGSGEVDYKNDRSPVVSDSVSFHSLSPIDGPPSNGPSKSPESTNLDDSVKSRPDTDAP 816
H sapiens HSASPTLGFAMMASLDAPGKVGPAPFNLQRQGSRENGSVE...SDGLTNDSSSLMGDQEYQS.RSPDILETTSFQALSPANSQAESIKSKSPDAGSKAESSENS...... 815
M musculus HLASPSLGFSVLASLDTQGKGALPALALQRQSSRENSSLEG.DTGPANDSSLLVGDQECQS.RSPDATETMCYQAVSPANSQAGSVKSRSP.EGHKAEGVESC...... 823
consensus
logo
X tropicalis QN..GSLNPDGDGALDLTNGGFTRK.IKEEPGL.LQNGELGRS..PNMYVGAPPALIKMEVSSD 852
G gallus ES..APGEMEGVVALDLTYGNIGRKVIKEEPGLHFANGEYGRNSIPAAFVRAPPALIKMEIPSE 889
A mexicanum EHRPMAQFFGNPMDRPLDRSLGNYMDHPVGNSIDRPAESELDDSMENSMDQSMYPDTDGALDLTSANLGRRVIKEEPGLYFSNGDYGRSSASGAFLQAPPPLVKLEASSD 915
D rerio QD...FSENNGALDLTSS.FTSKPIKEEPGLSFASGEYGSS..QLPFMRVPPSLVKLEMQIP 872
H sapiens ....RTEMEGRSSLPSTFIRAPPTYVKVEVPG. 843
M musculus ....RVDTEGRTSLPPTFIRAQPTFVKVEVPG. 851
consensus
logo
X tropicalis RMAG.....AAQFLGPPNLSPGLTPLIVPQR...RSAKQHICHTCGKNFSSASALQIHERTHTGEKPFACTICGRAFTTKGNLKVHVGTH.MWNNSARRGRRLSVDNQIP 953
G gallus RPIS.....TSHFIGPPALSPGVAPLLVPRPKFCRPAKQHICTTCGKNFSSASALQIHERTHTGEKPFACTICGRAFTTKGNLKVHVGTH.MWNNSARRGRRLSIDNPMA 993
A mexicanum RLFGDLHTGAMQLGSQPPASSRLSPLLAPPR...RSAKQHICTTCGKNFSSASALQIHERTHTGEKPFACTICGRAFTTKGNLKVHVGTH.MWNNAARRGRRLSIDNPTT 1021
D rerio PETP....MGAHGLYSSQLPQGAATPPASSSAPRRSTKQHLCNMCGKNFSSASALQIHERTHTGEKPFACSICGRAFTTKGNLKVHVGTH.MWNNTARRGQRLSLDNPMA 977
H sapiens ...........TFVGPSTLSPGMTPLLAAQP..RRQAKQHGCTRCGKNFSSASALQIHERTHTGEKPFVCNICGRAFTTKGNLKVHYMTHGANNNSARRGRKLAIENTMA 940
M musculus ...........TFVGPPSMPSGMPPLLASQPQPRRQAKQHCCTRCGKNFSSASALQIHERTHTGEKPFVCNICGRAFTTKGNLKVHYMTHGANNNSARRGRKLAIENPMA 950
consensus

logo

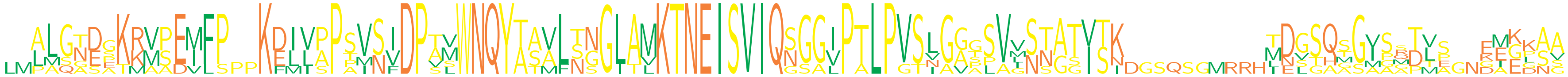

```
X tropicalis  ..ALGTDAKKVAEIFP..KFIVPPTVSIDPTMWNQYATALSNGLALKTNEISVIQNSAVPALPVSNGVAPVLSTATVTN..........IDVSAASVAQTLP..EIGDSA  1047
G gallus      ..LLGNDPKKVSEMFP..KDIVAPSVSIDPTVWNQYTAVLSNGIAMKTNEISVIQSGGLPTLPVTVAGGSAINTATVSK..........TDGTQSGAGSDTE..KAGGAA  1087
A mexicanum   ..PAQGSETRMPEMFP..KDMVPPSMSIDPALWNQYAAVLTSGLAMKTNEISVIQGGGIPTLPVSIGGGSVMSSASITIDGSQSGMRRHTNLGHGGMSRTLS..DMEKAS  1125
D rerio       LMAMGNESKMMPDMLPPPKDLIPPPINFDPSMWNQYAAAFTNGLTMKTNEISVIQNGGIP.LPGSLGGPLVGSTGGITK..........MESSQMGIPGTVA..EMEKNG  1074
H sapiens     ..LLGTDGKRVSEIFP..KEILAPSVNVDPVVWNQYTSMLNGGLAVKTNEISVIQSGGVPTLPVSLGATSVVNNATVSK..........MDGSQSGISADV....EKPSA  1032
M musculus    ..ALSAEGKRAPEVFS..KELLSPAVSVDPASWNQYTSVLNGGLAMKTNEISVIQSGGIPTLPVSLGASSVVSNGTISK..........LDGSQTGVSMPMSGNGEKLAV  1046
consensus        ** * *** ****  !*** !****!! *!!!!  *** !***!!!!!!!!! ***!*!!***** **********          ** ** **   *        *
```

logo

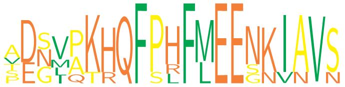

```
X tropicalis  AESVPKHQFSHFMEEN.IAVN.  1067
G gallus      ADNVPKHQFPLFMEENKIAVS.  1108
A mexicanum   VDNLQTRQFPRFMEESNIAVS.  1146
D rerio       SESIAK..FPHFMEEGKVN...  1091
H sapiens     TDGVPKHQFPHFLEENKIAVS.  1053
M musculus    PDGMAKHQFPHFLEENKIAVS.  1067
consensus      * * ***!**!*!!******
```
