# Supplementary figures and images for: A novel role for SALL4 during scar-free wound healing in axolotl
Source: NPJ Regen Med. 2016 Dec 8;1:16016–. doi: 10.1038/npjregenmed.2016.16 (PMC5612448; doi:10.1038/npjregenmed.2016.16)

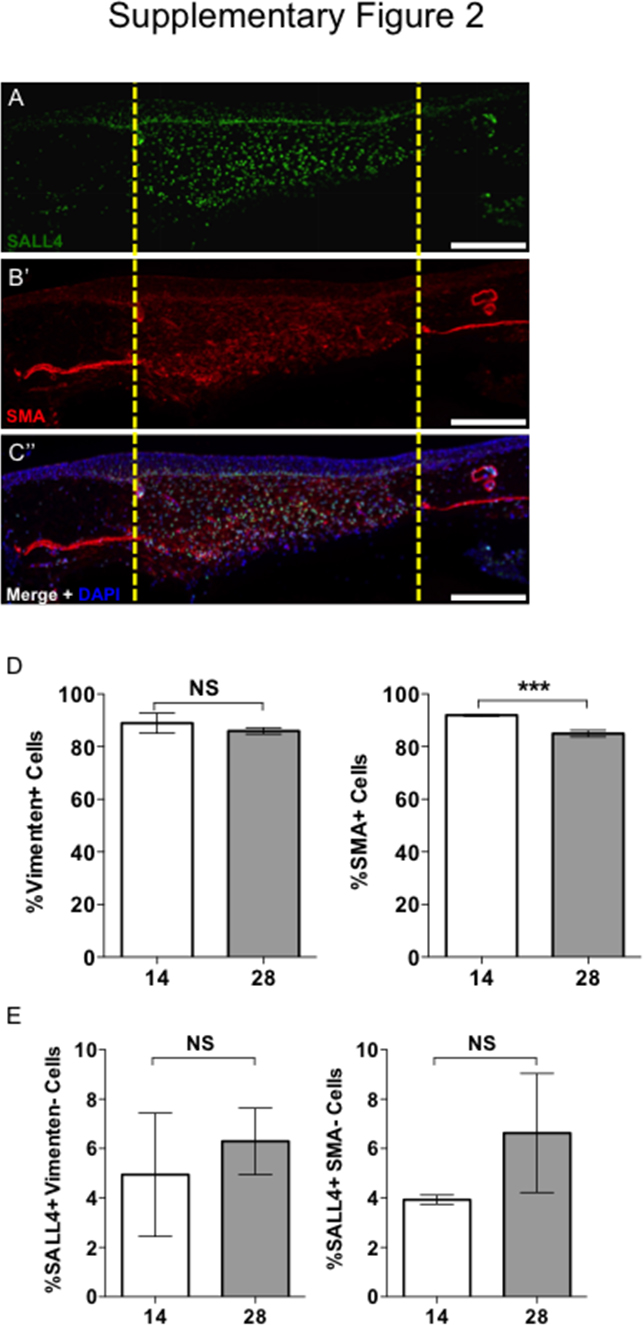

Supplement: Supplementary Figure 2 [file npjregenmed201616-s2.jpg]

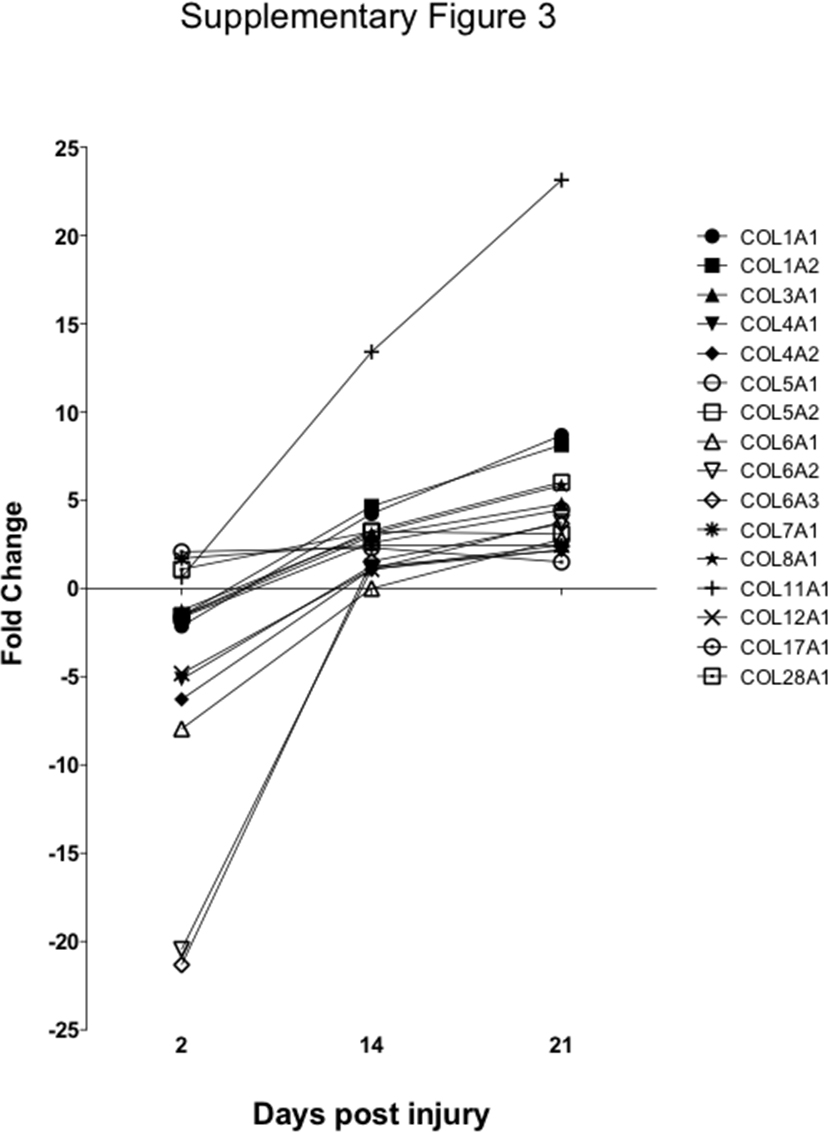

Supplement: Supplementary Figure 3 [file npjregenmed201616-s3.jpg]

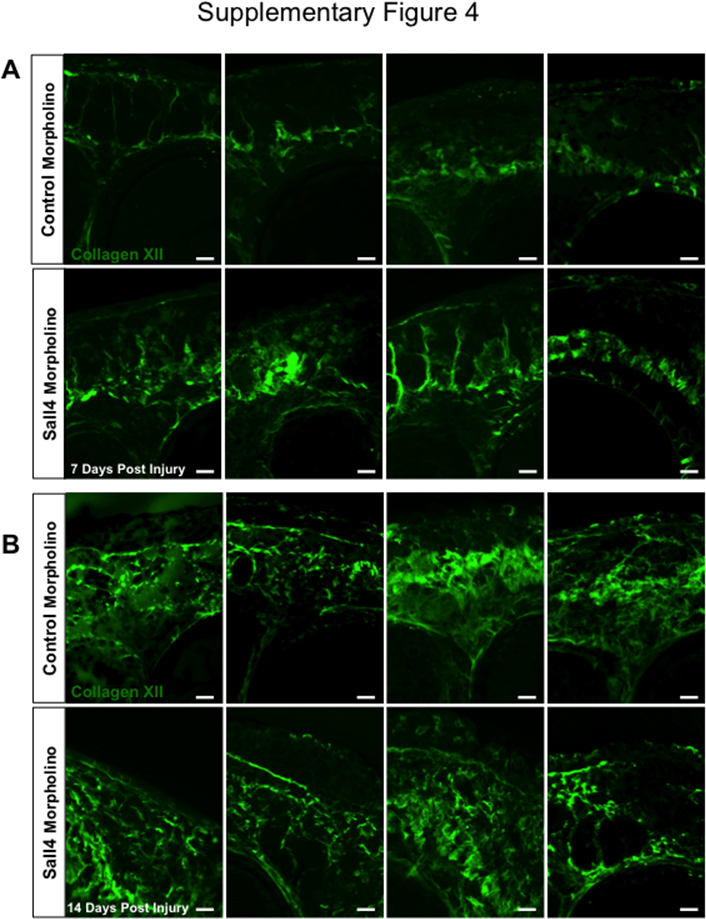

Supplement: Supplementary Figure 4a [file npjregenmed201616-s4.jpg]

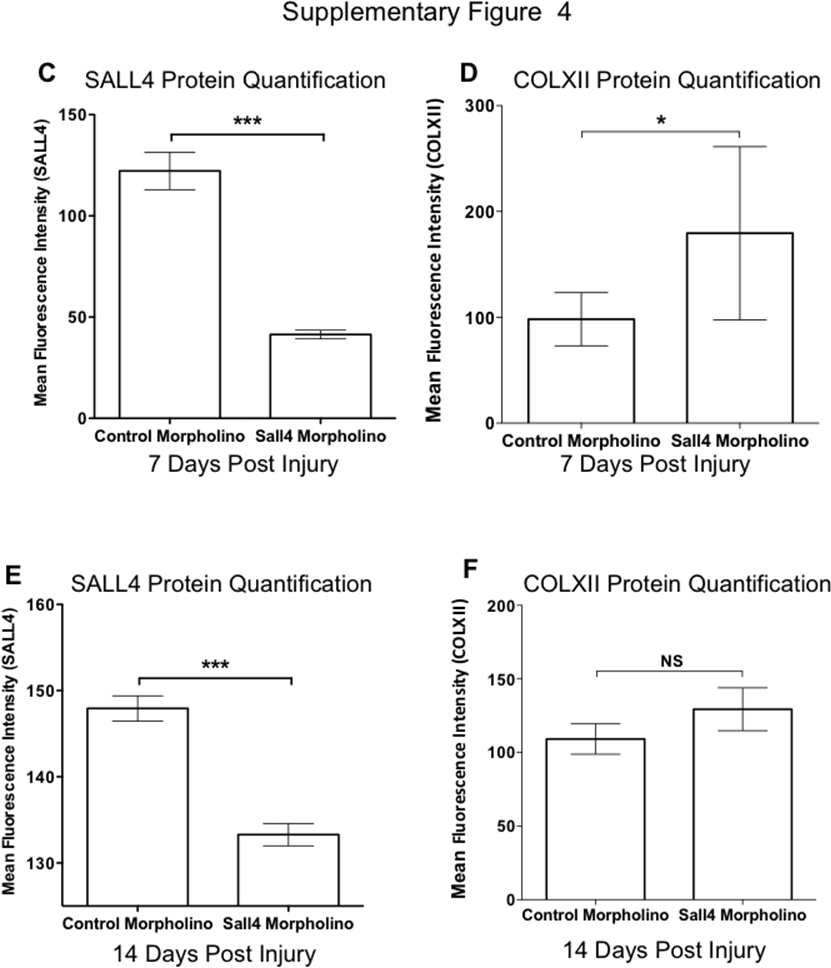

Supplement: Supplementary Figure 4b [file npjregenmed201616-s5.jpg]

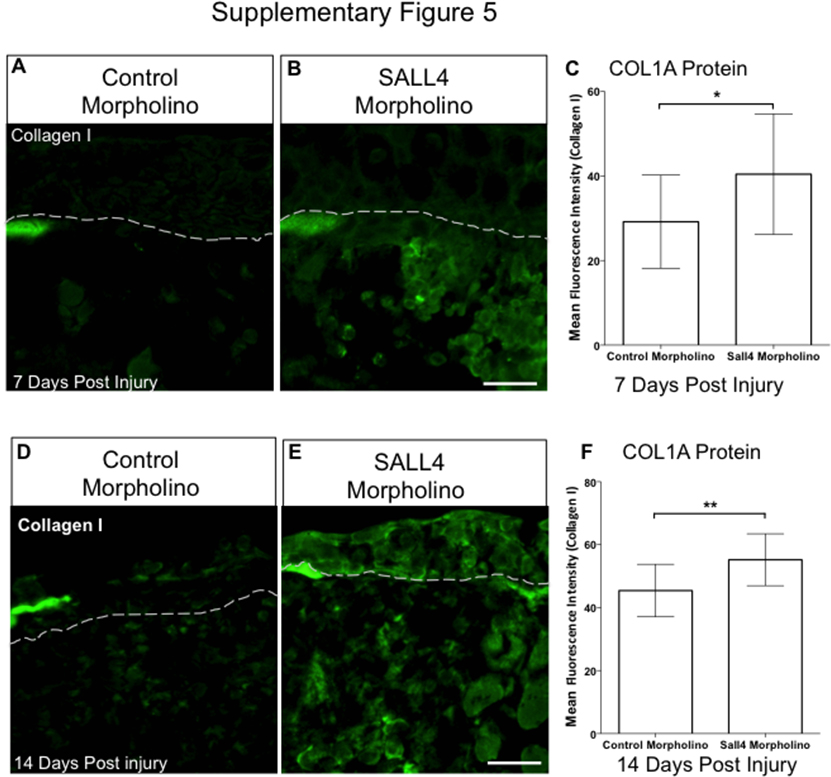

Supplement: Supplementary Figure 5 [file npjregenmed201616-s6.jpg]

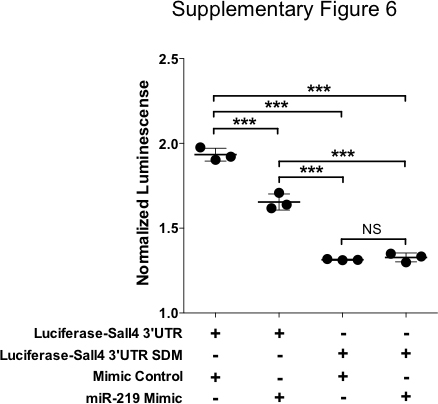

Supplement: Supplementary Figure 6 [file npjregenmed201616-s7.jpg]
